# Parental Psychological Control and Risk-Taking among Taiwanese Adolescents and Emerging Adults: Benefit Perception as a Mediator

**DOI:** 10.3390/ijerph21091207

**Published:** 2024-09-13

**Authors:** Catherine P. Chou

**Affiliations:** Department of Psychology, National Taiwan University, Taipei 10617, Taiwan; cpwchou@ntu.edu.tw

**Keywords:** psychological control, risk-taking, benefit perception, adolescent, emerging adult

## Abstract

Youth risk-taking behaviors present important public health concerns due to their prevalence and potential adverse consequences, underscoring the need for research and prevention strategies to promote youth’s healthy development. The present research examined the relationship between parental psychological control and risk-taking behaviors via benefit perception among high school and college students in Taiwan. Using a cross-sectional design, the study surveyed 378 participants to assess maternal and paternal psychological control, benefit perception, and engagement in risk-taking behaviors. Results indicated no significant difference in psychological control or benefit perception between high school and college students. However, college students reported higher levels of risk-taking behaviors, such as risky driving, alcohol use, and unprotected sex. Both maternal and paternal psychological control positively correlated with benefit perception and risk-taking behaviors. Furthermore, benefit perception mediated the relationship between psychological control and risk-taking behavior among high school and college students. These findings suggested that parental psychological control indirectly influenced youth risk-taking by shaping their perceptions of the benefits of such behaviors. The study highlights the importance of promoting autonomy-supportive parenting to reduce risk-taking behaviors and advocates for programs that enhance decision-making skills among adolescents and emerging adults.

## 1. Introduction

Adolescents and emerging adults, collectively referred to as youth, experience significant physical, cognitive, and psychosocial changes during this life stage [1]. They are more likely than other age groups to engage in risk-taking behaviors because of heightened sensitivity to rewards, peer influences, and ongoing brain development in regions associated with decision-making and impulse control [2,3,4]. Risk-taking involves engaging in behaviors that carry potential negative consequences [5]. Among youth, common health-related risk-taking behaviors include reckless driving, tobacco use, alcohol use, and drug use [6].

Youth risk-taking behaviors present important public health concerns, given the alarming rate of young individuals engaging in such behaviors worldwide. For example, according to the 2022 Taiwan Youth Health Survey [7], only 24.3% of high school students in Taiwan always wear seat belts when riding in the back seat of a car. Additionally, 25.6% have smoked cigarettes at some point, and 30.6% having consumed alcohol in the past 30 days. These behaviors can have profound implications for youth’s immediate and long-term health, such as addiction, chronic health issues, and increased risk of injury or death [8]. The prevalence and consequences of risk-taking behaviors among youth underscore the need for research and intervention to promote healthy development and prevent adverse outcomes.

Parental psychological control, a form of intrusive parenting that manipulates the child’s psychological and emotional state [9], has been associated with a range of negative outcomes in adolescents, including increased risk-taking behavior [10,11]. However, the mechanisms through which parental psychological control influences risk-taking remain underexplored. One potential mediator is benefit perception, an individual’s belief about the positive outcomes and advantages expected to gain from engaging in a specific behavior [12], which has not been thoroughly examined in relation to psychological control. Therefore, the purpose of the current study was to explore these relationships among Taiwanese high school and college students, contributing to a more comprehensive understanding of the impact of parental psychological control on youth’s risk-taking behavior.

### 1.1. Psychological Control

Parental psychological control is a parenting practice characterized by intrusive and manipulative behaviors directed at children’s thoughts and feelings [9]. It involves behaviors that intrude upon children’s psychological world, such as emotional manipulation and pressure to ensure compliance with parental expectations [13]. Parents employing psychological control may restrict verbal expressions, invalidate their children’s feelings, engage in personal attacks, induce guilt, withdraw affection, show disrespect, and use shaming tactics [9,14]. This approach differs from behavioral control, which focuses on setting clear rules and monitoring children’s behavioral world [15].

Psychological control has been consistently linked to negative effects on youth development (e.g., [15,16]), as parental attempts to manipulate children’s feelings and thoughts through psychological means can hinder independence and autonomy in their children. This form of control has been associated with increased negative emotions [17], depression [18], social anxiety [19], low self-esteem [20], and poor psychological well-being [21]. Additionally, the negative impact of parental psychological control on adolescents can persist into adulthood, particularly in the Chinese context [22].

Research indicated that Chinese parenting is frequently characterized by an authoritarian and restricting style [23,24]. Chinese parents are likely to use psychological control, which can be attributed to Confucianism’s emphasis on collectivity, hierarchy, and a sense of obligation [25,26]. Within this cultural context, children are expected to respect their parents’ demands and show obedience [27,28]. Consequently, Chinese parents often exert psychological control to reinforce these cultural values of respect for parents, conformity, and order. Despite the frequent use of psychological control by Chinese parents, the impact on their children might be smaller than on American children (e.g., [25,29]). Thus, an empirical investigation on psychological control in the Taiwanese context is necessary.

#### Psychological Control and Risk-Taking

Research investigating the relationship between psychological control and risk-taking behaviors has yielded mixed results. Some studies suggest that psychological control undermines attachment security, potentially fostering aggressive and norm-violating behavior [30]. Indeed, several studies have found a positive association between parental psychological control and adolescents’ aggressive and externalizing behaviors [31,32,33]. A meta-analysis by Pinquart (2017) [34] confirmed that psychological control was linked to higher levels of externalizing problems and could predict changes in these problems over time. Similarly, Yan et al. (2020) [35] found a significant positive association between parental psychological control and both externalizing and internalizing problem behaviors in youth. However, other studies reported no significant relationship between psychological control and adolescent delinquency or externalizing behaviors [15,36,37]. Some research indicates that psychological control may have a more pronounced impact on the onset of internalizing problems rather than externalizing behaviors [38,39]. Therefore, the relation between psychological control and risk-taking behavior needs further examination.

Some researchers argue that parental psychological control may indirectly influence youth risk-taking behaviors. For instance, Bai et al. (2020) [40] indicated that self-control and need frustration mediated the association between parental psychological control and externalizing behaviors among Chinese adolescents. In addition, Inguglia et al. (2022) [41] suggested that the relationship between parental psychological control and drinking behavior was mediated by adolescents’ coping strategies. Another study by Cui et al. (2014) [42] demonstrated that parental psychological control indirectly affected adolescent aggressive behavior through its impact on emotion regulation. These findings indicate that parental psychological control can influence youth risk-taking behaviors both directly and indirectly.

### 1.2. Benefit Perception

Benefit perception refers to an individual’s assessment of the positive outcomes that may result from a specific action or behavior [12]. This concept plays a crucial role in decision-making processes. Reyna and Farley (2006) [43] explained that youth are more likely to engage in high-risk behaviors because they place greater weight on potential benefits than on perceived risks when making decisions. Perceived benefits seem to be important determinants of youth’s risk-taking intentions [44].

The association between benefit perception and risk-taking have been demonstrated in studies across various behaviors. For example, White et al. (2006) [45] indicated that individuals were more likely to use party drugs if they perceived benefits of drug use, such as mood effects and social advantages. Moreover, Goldberg et al. (2002) [46] found that perceptions of benefits were significantly related to drinking and smoking six months later. Another study showed that adolescents’ perceived benefits were strongly associated with their reported involvements in risk-taking behaviors, such as alcohol use, illegal drug use, and sexual activity [47]. It is suggested that youth’s perceived benefits of immediate gratification and peer acceptance often outweigh concerns about long-term health risks, leading to increased risk-taking behaviors [48].

#### Benefit Perception as a Mediator

Previous studies have found an association between parental control and youth decision-making. For instance, Pérez and Cumsille (2012) [49] suggested that adolescents’ perceptions of maternal psychological control and paternal psychological control were linked to their decision-making in prudential domain (e.g., alcohol and drug use) and personal domain (e.g., hairstyle), respectively. Furthermore, Grolnick et al. (1997) [50] indicated that perceived parental control could restrict adolescents’ ability to make decisions and decrease their behavioral autonomy, particularly when parental control emphasizes obedience and conformity to societal and familial standards. As a result, parental control can influence one’s decision-making through internalization, fostering the development of certain decision-making styles in children and adolescents [51,52].

According to self-determination theory [53], autonomy, competence, and relatedness are important in fostering healthy psychological development and well-being. When parents exert psychological control, it impedes youth’s desires for self-determination and autonomy. This control is internalized by young individuals, influencing their decision-making processes in a way that may lead them to emphasize the perceived benefits of risk-taking behaviors as a means to reclaim some control over their choices. Consequently, psychological control is thought to be internalized into the decision-making process through benefit perception, which in turn influences their propensity for engaging in risk-taking behaviors. The current study, therefore, proposed that benefit perception mediates the relationship between parental psychological control and youth risk-taking.

### 1.3. Developmental Changes

The transition from high school to college represents an important developmental milestone, as individuals move from adolescence to emerging adulthood, offering significant opportunities for personal growth and behavioral change. Adolescence involves increased cognitive capabilities, such as decision-making skills, while risk-taking behavior is often heightened due to the ongoing maturation of the prefrontal cortex [54]. Emerging adulthood, generally viewed as ages 18 to 25, is a transitional phase where individuals gain greater independence and self-sufficiency [1]. Unlike adolescence, emerging adulthood is characterized by more autonomy and self-directed decision-making, yet it continues to be a time of identity exploration and instability as individuals navigate the challenges of adulthood [55].

According to research, parents often maintain a degree of control over their children well into emerging adulthood [56]. For example, studies have found associations between parental control and emerging adults’ behaviors and emotions [57,58,59,60]. These findings imply that the influence of parental control extends beyond adolescence into emerging adulthood. However, there might be developmental changes, such that individuals may perceive a decline in their parents’ communication as they enter emerging adulthood [61].

Similarly, decision-making processes also evolve from adolescence to emerging adulthood. Christakou et al. (2013) [62] studied the maturation of decision-making using fMRI, showing that adolescents and young adults engaged in distinct neural and psychological processes during decision-making. Reyna and Farley (2006) [43] proposed that individuals become more risk-averse as they develop, with adults being better equipped to process contextual information accurately compared to adolescents [63]. Another study further indicated that adolescents might exhibit less decision-making competence than adults in evaluation processes [64]. This suggests that decision-making, specifically benefit perception, may differ between high school and college students.

Moreover, research suggested that several risk-taking behaviors increase in emerging adulthood and decline into adulthood [65]. A study by Fromme et al. (2008) [66] found that the transition from high school to college led to increased alcohol consumption, marijuana use, and sexual activity with multiple partners. The increased engagement in risk-taking behavior could be attributed to decreased adult supervision, increased personal freedom, and greater availability of substances through peers and social activities, as many college students no longer reside at home with their parents.

Prior research indicates that developmental changes in cognition, behavior, and relationships with parents occur as individuals transition from adolescence to emerging adulthood. Comparing high school and college students in terms of their perceived parental control, benefit perception, and risk-taking behaviors can be informative in exploring developmental shifts.

### 1.4. Current Study

The purpose of the present research was to examine the relationship between parental psychological control and risk-taking behaviors via benefit perception among high school and college students in Taiwan. The study aimed to contribute to the existing literature by addressing several gaps. First, there has been limited research on how psychological control by parents is internalized into youth’s decision-making process, specifically benefit perception, thereby influencing risk-taking behavior. Second, the study utilized a cross-sectional design to compare high school and college students, an approach that is relatively uncommon in the psychological control literature, particularly within the Taiwanese context. Third, the study included both maternal and paternal control. While the active role of fathers in child-rearing is increasingly recognized, most studies on parental control have focused solely on maternal control (e.g., [10,67]). Analyzing maternal and paternal control independently enables an assessment of whether the pathways through which parental psychological control influences youth risk-taking behavior are consistent across both parental figures or if they diverge.

#### 1.4.1. Specific Aims

The specific aims of the study were as follows: (1) To assess the levels of parental psychological control, benefit perception, and risk-taking behaviors among high school and college students in Taiwan; (2) To examine the associations between parental psychological control, benefit perception, and risk-taking behaviors; and (3) To investigate the mediating role of benefit perception in the relationship between psychological control and risk-taking behavior.

#### 1.4.2. Hypotheses

Based on the literature review, the study had the following hypotheses. First, it was hypothesized that high school students would perceive higher levels of psychological control and benefit perception than college students, while college students would engage in more risk-taking behavior than high school students. Second, psychological control was predicted to be positively correlated with both benefit perception and risk-taking behavior. Benefit perception would also have a positive correlation with risk-taking behavior. Third, the relationship between psychological control and risk-taking behavior was expected to be mediated by benefit perception. No specific hypothesis was made regarding the difference between maternal and paternal psychological control and their relationships with benefit perception and risk-taking behavior.

## 2. Materials and Methods

### 2.1. Participants

A total of 378 high school and college students in Taiwan participated in the study. Descriptive statistics of their demographics are shown in Table 1. All participants were of Taiwanese descent and fluent in Mandarin. For the high school sample, 222 students (150 males, 69 females, 3 prefer not to say) from two high schools in Taipei were recruited. These participants were first- or second-year senior high school students, with an age range of 15–19 (M = 16.18, SD = 0.70). Most (97.3%) lived with their parents/family at home.

For the college sample, 156 students from 39 different universities/colleges across 21 cities were recruited online. This group included 54 males and 102 females, with an age range of 18–20 (M = 18.94, SD = 0.62). The majority (91.7%) were in their first or second year of college. Regarding residence, 42.3% lived on campus, 27.6% lived at home, and 30.1% lived off campus alone or with roommates.

### 2.2. Design and Procedure

Two senior high schools were selected using convenience sampling. After obtaining permission from school teachers to conduct the research, informed consent was collected from both students and their parents. Students then completed a paper survey during regular class time. For the college sample, the study was advertised on a nationwide online platform for Taiwanese college students. Interested participants received a link to an online survey hosted on SurveyMonkey.com after signing up for the study. The online survey began with an informed consent form that participants reviewed before proceeding to the survey questions. The survey took an average of 40 min to complete. Upon completion, participants received a TWD 100-dollar gift card as compensation. The study protocol was reviewed and approved by the affiliated Institutional Review Board.

### 2.3. Measures

This study was part of a larger research project investigating the factors that influence risk-taking. The current analyses focused on measures assessing perceived parental psychological control, benefit perception, and risk-taking behaviors. The survey materials were provided in Mandarin Chinese. Measures initially developed in English were translated into Mandarin Chinese and then back-translated by bilingual researchers. The original and back-translated versions were compared to identify and resolve any inconsistencies.

#### 2.3.1. Psychological Control

Parental psychological control was assessed using a combination of three scales: the 10-item Psychological Control Scale [68], the 8-item Psychological Control-Disrespect Scale [9], and one item measuring shame [14]. Psychological control consists of the following sub-domains: Constraining Verbal Expression (e.g., “My father is a person who often interrupts me”.), Invalidating Feeling (e.g., “My father is a person who is always trying to change how I feel or think about things”.), Personal Attack (e.g., “My father is a person who brings up my past mistakes when he criticizes me”.), Love Withdrawal (e.g., “My father is a person who will avoid looking at me when I have disappointed him”.), Guilt Induction (e.g., “My father is a person who tells me of all the things he had done for me”.), Disrespect (e.g., “My father is a person who ridicules me or puts me down (e.g., saying I am stupid, useless, etc.)”.), and Shame (e.g., “My father is a person who says, any behavior that brings shame to me also brings shame to my family”.).

When defining “parent”, participants were asked to include both biological and non-biological parents (e.g., stepfather). Maternal control and paternal control were rated separately. The items were scored on a 3-point Likert scale (0 = not like her/him to 3 = a lot like her/him). Higher scores indicated greater perceived levels of psychological control. The Cronbach’s alpha coefficients of internal consistency for maternal and paternal psychological control in the high school sample were 0.895 and 0.912, respectively, while in the college sample they were 0.889 and 0.919, respectively.

#### 2.3.2. Benefit Perception

Benefit perception was assessed using the revised perceived benefits subscale of the Risk Involvement and Perception Scale [44]. This subscale evaluates participants’ perceptions of how advantageous or beneficial it would be to engage in each of nine behaviors (e.g., drinking alcohol, smoking cigarettes). Participants rated on a 9-point scale, ranging from 1 = not at all beneficial to 9 = extremely beneficial. The Cronbach’s alphas were 0.899 and 0.885 for the high school and college samples, respectively.

#### 2.3.3. Risk-Taking Behavior

Risk-taking behavior was measured using the revised involvement subscale of the Risk Involvement and Perception Scale [44]. Participants reported their engagement in each of 14 different behaviors over the past three months, encompassing a wide range of risk activities, such as riding with a drunk driver and having unprotected sex. They rated their involvement on a 9-point scale, from 1 = never to 9 = daily. For this study, only 9 items related to risky driving, alcohol consumption, drug use, smoking, and unprotected sex were analyzed. The Cronbach’s alpha coefficients for these items were 0.833 for the high school sample and 0.701 for the college sample. Discriminant validity between risk-taking behavior and benefit perception was established by meeting the Fronell–Larcker criterion [69].

#### 2.3.4. Demographic Characteristics

Participants completed a questionnaire covering various demographic details. They were asked to provide information about their gender, age, school year, place of residence (e.g., on campus, at home, or off campus), and their parents’ marital status and education levels.

### 2.4. Analytical Strategies

Data analyses were conducted using SPSS 29.0 [70]. First, a one-way multivariate analysis of variance (MANOVA) and an independent t-test were performed to examine differences between the high school and college samples in the levels of the study variables. Second, zero-order correlations among the variables were computed by school level (i.e., high school and college). Third, the mediation model was tested. Model 4 in the PROCESS macro was used to test for indirect effects (10,000 bootstrapped samples) [71]. Analyses for maternal control and paternal control were conducted separately.

#### Missing Data

For parental psychological control, some participants only had one parent (refer to Table 1), providing perceived control from either the mother or father. For other study variables, missing values ranged from 0 to 0.8% and were missing completely at random (Little’s MCAR test: χ^2^(32) = 39.525, *p* = 0.169).

## 3. Results

### 3.1. High School vs. College in the Levels of Study Variables

The MANOVA results showed that there was no overall effect of school levels on maternal psychological control (Wilks’ λ = 0.986, F(7, 364) = 0.720, *p* = 0.655) nor paternal psychological control (Wilks’ λ = 0.970, F(7, 353) = 1.578, *p* = 0.141), as presented in Table 2. However, there was an overall effect of school levels on risk-taking behavior (Wilks’ λ = 0.865, F(5, 371) = 11.542, *p* < 0.001). The college sample engaged in more risky driving (F(1, 375) = 17.943, *p* < 0.001), alcohol use (F(1, 375) = 19.141, *p* < 0.001), and unprotected sex (F(1, 375) = 19.666, *p* < 0.001) than the high school sample, as displayed in Table 3. Independent t-test indicated that there was no difference in benefit perception between the high school and college samples (t(368) = 0.599, *p* = 0.549).

### 3.2. Correlations of Study Variables

Bivariate correlations of the study variables are presented in Table 4; Table 5. Results showed that in the high school sample, maternal psychological control (i.e., Invalidating Feeling, Love Withdrawal, Disrespect, Shame) was positively correlated with benefit perception (r = 0.142, 0.141, 0.220, and 0.212, respectively). Two forms of maternal psychological control (i.e., Disrespect, Shame) were positively correlated with risk-taking behavior (r = 0.197 and 0.167). Benefit perception also had a positive association with risk-taking behavior (r = 0.597).

Similar results were found in the college sample, such that maternal psychological control (i.e., Constraining Verbal Expression, Personal Attack, Love Withdrawal, Disrespect, Shame) was positively correlated with benefit perception (r = 0.295, 0.164, 0.187, 0.212, and 0.219, respectively). In terms of risk-taking behavior, it had positive associations with maternal psychological control (i.e., Constraining Verbal Expression, Invalidating Feeling, Guilt Induction, Love Withdrawal, Disrespect, Shame; r = 0.248, 0.176, 0.201, 0.321, 0.287, and 0.270, respectively) and benefit perception (r = 0.460).

Consistent with maternal control, in the high school sample, paternal psychological control (i.e., Constraining Verbal Expression, Invalidating Feeling, Love Withdrawal, Disrespect, Shame) was positively correlated with risk-taking behavior (r = 0.155, 0.137, 0.154, 0.225, and 0.232, respectively). One form of paternal psychological control (i.e., Love Withdrawal) was positively associated with benefit perception (r = 0.162).

In the college sample, paternal psychological control (i.e., Personal Attack, Love Withdrawal, Disrespect, Shame) was positively correlated with benefit perception (r = 0.321, 0.324, 0.277, and 0.165, respectively). Paternal psychological control (i.e., Personal Attack, Guilt Induction, Love Withdrawal, Disrespect) also had positive correlations with risk-taking behavior (r = 0.166, 0.205, 0.262, and 0.170, respectively).

### 3.3. The Hypothesized Mediation Model

The data from the high school and college samples were combined for the mediation model, given that there was no difference between the two samples in maternal psychological control, paternal psychological control, or benefit perception. Maternal and paternal models were tested separately. School level and gender were controlled. The unstandardized estimated path coefficients are presented in Table 6.

#### 3.3.1. Maternal Model

**Figure 1:**
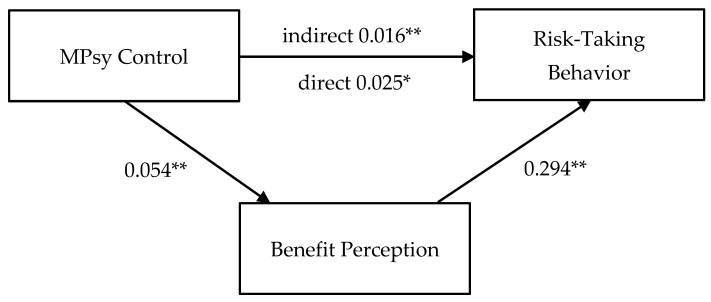
Maternal mediation model. Note: * *p* < 0.05; ** *p* < 0.01 (2-tailed). MPsy Control: maternal psychological control.

In the maternal model, benefit perception was hypothesized to mediate the relationship between maternal psychological control and risk-taking behavior when controlling school level and gender. Percentile bootstrap confidence intervals were calculated in PROCESS to test the statistical significance of mediation indirect effect (see Figure 1).

First, benefit perception was regressed on maternal psychological control. This regression equation was significant (F(3, 356) = 6.104, *p* < 0.001, R^2^ = 0.049), such that participants with a higher level of perceived maternal psychological control were more likely to perceive benefits in risk-taking situations (b = 0.054, *p* = 0.001).

Second, risk-taking behavior was regressed on maternal psychological control and benefit perception. This regression equation was also significant (F(4, 355) = 31.452, *p* < 0.001, R^2^ = 0.262). Controlling for school level and gender, higher benefit perception predicted more risk-taking behavior (b = 0.294, *p* < 0.001).

The results revealed a significant indirect effect of maternal psychological control on risk-taking behavior (b = 0.016, 95% CI [0.006, 0.032]), suggesting that risk-taking behavior is influenced by maternal psychological control through a sequence in which participants who perceived higher maternal psychological control were more likely to perceive benefits, which, in turn, increased their engagement in risk-taking behavior. Because the direct effect of maternal psychological control on risk-taking behavior in the presence of the mediator was also significant (b = 0.025, *p* = 0.022), this was a partial mediation effect.

#### 3.3.2. Paternal Model

**Figure 2:**
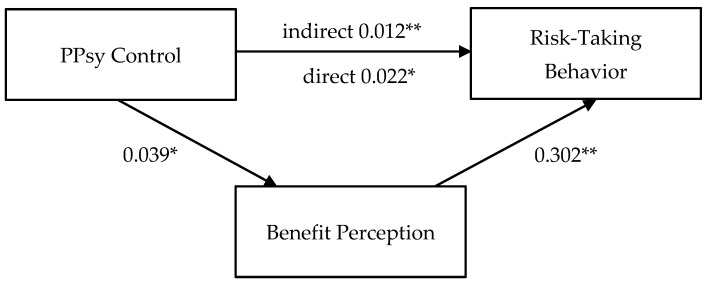
Paternal mediation model. Note: * *p* < 0.05; ** *p* < 0.01 (2-tailed). PPsy Control: paternal psychological control.

The same procedures using PROCESS were performed to examine the paternal model (see Figure 2). Paternal psychological control had a significant effect on benefit perception (F(3, 345) = 4.370, *p* = 0.005, R^2^ = 0.037), such that participants with higher paternal psychological control were more likely to perceive benefits in risk-taking situations (b = 0.039, *p* = 0.029). In addition, risk-taking behavior was regressed on paternal psychological control and benefit perception. This regression equation was significant (F(4, 344) = 31.705, *p* < 0.001, R^2^ = 0.269). Higher benefit perception predicted more risk-taking behavior (b = 0.302, *p* < 0.001). Moreover, the indirect effect of paternal psychological control on risk-taking behavior was significant (b = 0.012, 95% CI [0.002, 0.028]), while the direct effect was still significant (b = 0.022, *p* = 0.047), suggesting a partial mediation effect.

## 4. Discussion

The purpose of the current study was to explore the relationship between parental psychological control and risk-taking behaviors as mediated by benefit perception among high school and college students in Taiwan. The findings partially supported the hypotheses and are discussed below.

### 4.1. Developmental Changes

The hypothesis that college students would engage in more risk-taking behavior than high school students was supported. Results showed that college students engaged in more risky driving, alcohol use, and unprotected sex, compared to high school students. These findings align with the prior studies showing that emerging adults are more inclined to engage in risk-taking behavior than younger or older individuals [65,66].

The increase in risk-taking behaviors from adolescence to emerging adulthood may be attributed to youth’s identity formation. Adolescents engage in risk-taking as a way of exploration and gaining excitement, whereas emerging adults are more prone to take risks due to unclear social expectations and ongoing identity formation [72,73]. Engaging in risky activities, such as alcohol use and sexual behavior, may be perceived by emerging adults as part of adopting adult roles [74]. Therefore, risk-taking behaviors peak among emerging adults as they define their social roles [75].

Surprisingly, the hypothesis that high school students would perceive a higher level of psychological control than college students was not supported. This finding is inconsistent with Arnett’s (2015) [55] suggestion that parent–child relationships in emerging adulthood often shift from conflict to companionship. One possible explanation could be that the age range of the college sample in the present study was 18–20 years old. Students during their first two years in college might still view themselves as adolescents. Additionally, parents may continue to exert psychological control over their college-aged children, especially in the Taiwanese context, which emphasizes family cohesion and obedience [14]. As a result, no difference was found between adolescents and emerging adults in terms of parental psychological control.

Similarly, the study found no difference between high school and college students in benefit perception, which contradicts prior research demonstrating that individuals place less weight on potential benefits as they age [43]. Neuroscientific research indicates that the frontal lobes, responsible for decision-making, continue to develop into late adolescence and early adulthood [76]. Due to the immaturity of the frontal lobes and the activation of pleasure-related brain areas when immediate rewards are present [77], both high school and college students in our sample were likely to base decisions on perceived benefits and short-term rewards. Consequently, adolescents and emerging adults in the present study showed no significant difference in benefit perception in their decision-making process.

### 4.2. Psychological Control and Risk-Taking

The hypothesis that psychological control would be positively correlated with risk-taking behavior was supported for both maternal and paternal control across high school and college samples. These findings are consistent with previous studies indicating that psychological control is linked to externalizing problems, rule-breaking, and deviant behaviors in both individualistic and collectivist cultures [33,78,79].

According to Georgiou and Symeou (2018) [80], youth may perceive parental psychological control as intrusive and inappropriate, particularly as they strive for increased autonomy and independence. This perception can drive them to engage in risk-taking behaviors as a means of escaping such control. For instance, the present study found that perceived disrespect from either a mother or father was significantly correlated with risk-taking behavior in both high school and college samples. The lack of respect toward youth’s individuality may hinder their abilities to self-express [35], leading to risk-taking behaviors, such as alcohol drinking [41], as a coping mechanism to deal with psychologically controlling parents.

### 4.3. Benefit Perception and Risk-Taking

In line with the prior research [46,47], the results showed that benefit perception was positively correlated with risk-taking behavior in both high school and college samples. In the context of risky decision-making, individuals go through several cognitive processing steps: identifying potential options, recognizing possible outcomes, evaluating the desirability of these outcomes, estimating their likelihood, and making a decision [81]. When youth perceive benefits in a behavior, they are likely to engage in that behavior due to the anticipated rewards serving as positive reinforcement. The perceived benefits may include immediate pleasure, excitement, social acceptance, and enhanced self-esteem. In addition, youth tend to have optimistic bias, which is the tendency to believe that they are less likely to experience negative consequences such as accidents, illnesses, and misfortunes compared to others [1]. This bias leads them to engage in risky activities despite the potential for adverse consequences [82], consistent with the suggestion by Siegel et al. (1994) [47] that perceived benefits are a more powerful predictor of adolescents’ risk involvement than perceived risks.

The association between benefit perception and risk-taking suggests that the affective system plays a dominant role in youth’s risky decision-making [47]. Risk-taking behavior is often influenced more by emotional and immediate concerns rather than cognitive and long-term considerations. Therefore, young individuals’ benefit perception dominates their decision-making, resulting in their risk-taking behavior. This relationship is evident across various domains, including social, ethical, gambling, recreational, and investment risk-taking [83].

### 4.4. Mediating Role of Benefit Perception

The mediating role of benefit perception in the relationship between psychological control and risk-taking behavior was confirmed. The indirect effects from psychological control to risk-taking behavior via benefit perception were found in both maternal and paternal control among high school and college students. This suggests that parental psychological control shapes how young individuals perceive the benefits of risk-taking behaviors, which in turn influences their likelihood of engaging in such behaviors.

The findings align with previous research demonstrating that parental psychological control indirectly influences risk-taking behaviors [40,41,42]. Parenting practices impact the development of decision-making skills, which are crucial in risk assessment and behavior [49]. The current study extends the existing literature by highlighting the critical role of benefit perception in the decision-making process, which in turn affects youth’s risk-taking behavior.

Psychological control has been shown to undermine youth’s self-esteem and independence [84]. When parents employ psychological control, youth may feel that their thoughts and opinions are invalidated. This can prompt them to focus more on the perceived benefits of their actions as a way to affirm their own ideas and decisions. That is, youth may focus on the immediate rewards (e.g., pleasure and social acceptance) that the behaviors might bring, subsequently engaging in risk-taking behaviors, such as sexual intercourse and alcohol use, to regain a sense of autonomy and self-worth [67].

Self-determination theory [53] provides a framework to understand these findings. The theory posits that individuals have fundamental psychological needs for autonomy, competence, and relatedness. Parental psychological control disrupts these needs by imposing standards on young people’s thoughts and feelings. Bai et al. (2020) [40] indicated that such control, including compelling youth to conform to their parents’ expectations, making them feel insufficient in meeting these demands, and giving youth conditional love, hinders their basic psychological needs. By focusing on the perceived benefits of risk-taking behaviors, youth attempt to fulfill their basic psychological needs to assert their independence and validate their self-worth. This decision-making process leads young individuals to engage in risk-taking behaviors more frequently.

It is important to note that there was no significant difference between maternal and paternal psychological control in their effects on benefit perception and risk-taking behaviors. This finding indicates that both maternal and paternal influences are equally impactful in shaping youth’s perceptions and behaviors. Previous research has similarly noted that both parents play critical roles in their children’s development [85]. Both parents are important social agents in influencing young people cognitively and behaviorally across adolescence and emerging adulthood.

Moreover, it is noteworthy that benefit perception played a mediating role in the relationship between psychological control and risk-taking in the whole sample, including both high school and college students. Despite the lack of difference in psychological control and benefit perception between high school and college students, there was a notable difference in risk-taking behavior. This disparity can be attributed to the place of residence. In fact, the overall effect of residence place on college students’ risk-taking behavior was significant (Wilks’ λ = 0.793, F(15, 1107) = 5.913, *p* < 0.001). Specifically, risky driving, alcohol use, and unprotected sex were significantly more prevalent among students living on campus or off campus alone or with roommates, compared to those living at home. College students living away from home experience greater independence and freedom, coupled with reduced parental supervision, which provides more opportunities for engaging in risk-taking behaviors [55]. Thus, the physical context of residence might play a role in facilitating risk-taking behaviors, which needs further examination in future research.

### 4.5. Limitations and Future Direction

There are some limitations in the current study. First, the cross-sectional design limits the ability to draw causal inferences about the relationships between psychological control, benefit perception, and risk-taking behaviors. Longitudinal research, by tracking changes in these variables across different developmental stages, could provide deeper insights into their directionality and the dynamic interplay over time. Second, self-reported data may lead to bias due to social desirability or inaccurate recall. Future research could incorporate multiple data sources, such as parental reports and behavioral observations, to enhance the validity of the findings. Third, the study sample was drawn from a specific cultural context, namely Taiwan, which may limit the generalizability of the results to other cultural settings. Comparative studies across diverse cultural contexts are needed to explore potential cultural differences. Lastly, while this study focused on psychological control, other parenting dimensions, such as behavioral control and parental warmth, are also associated with risk-taking behavior. Future research could investigate how different parenting styles interact to influence youth’s risk-taking behaviors.

## 5. Conclusions

In conclusion, this study highlights the significant role of parental psychological control in influencing youth’s risk-taking behaviors through the mediation of benefit perception. Both high school and college students were similarly influenced by maternal and paternal psychological control in their perceptions of the benefits associated with risk-taking. These findings have several practical applications and implications for intervention.

From a practical standpoint, the results suggest that interventions could focus on promoting healthy parental practices that minimize psychological control. Parenting programs could emphasize the importance of autonomy-supportive parenting, which respects youth’s thoughts and feelings and fosters a sense of independence and competence. This approach can reduce the tendency towards risk-taking behaviors by supporting the psychological needs of young individuals. Educational settings can also benefit from these insights. Integrating decision-making skills into the curriculum can help youth critically assess the risks and benefits of their actions. Programs that enhance self-worth without reliance on risk-taking behaviors could further mitigate these tendencies. Additionally, universities could consider developing support systems to help students navigate the transition to adulthood without resorting to risk-taking behaviors, especially for those living away from home.

The findings from the present study suggest a multifaceted approach to reducing youth’s risk-taking behaviors, combining parental education and youth empowerment. Future research could continue to explore the dynamics of parental psychological control and risk-taking, with a focus on the role of decision-making processes, to prevent youth from engaging in risk-taking behaviors and promote positive developmental outcomes.

## Figures and Tables

**Table 1:** Descriptive statistics of the demographics. Note: * Junior college is one type of school in the Taiwanese education system.

Variables	High School	College
N	222	156
Male	150	54
Female	69	102
Age mean (SD)	16.18 (0.70)	18.94 (0.62)
Place of Residence		
Live on campus		42.3%
Live at home with parents/family	97.3%	27.6%
Live off campus (alone or with friends/roommates)		30.1%
Parental Marital Status		
Married	84.1%	84.6%
Separated/Divorced/Widowed	15.5%	14.8%
Never married/Single	0.4%	0.6%
Maternal Education Level		
Elementary/Junior high school	2.7%	17.4%
Senior high/Vocational high school	41.6%	40.6%
* 5-year junior college	20.1%	18.7%
College	27.4%	20%
Post graduate	7.7%	3.3%
Paternal Education Level		
Elementary/Junior high school	8.7%	9.7%
Senior high/Vocational high school	28.4%	40.0%
5-year junior college	22.5%	21.3%
College	26.1%	21.3%
Post graduate	12.4%	7.7%

**Table 2:** Levels of psychological control across school levels.

Variables	Maternal Control Mean (SD)		F	Paternal Control Mean (SD)		F
	High School	College		High School	College	
Psychological Control						
Constraining Verbal Expression	1.493 (0.562)	1.471 (0.539)	0.150	1.488 (0.563)	1.466 (0.597)	0.127
Invalidating Feelings	1.849 (0.766)	1.752 (0.737)	1.510	1.827 (0.742)	1.750 (0.755)	0.911
Personal Attack	1.829 (0.656)	1.788 (0.603)	0.379	1.535 (0.624)	1.537 (0.583)	0.001
Guilt Induction	1.728 (0.655)	1.631 (0.567)	2.228	1.552 (0.608)	1.412 (0.514)	5.200 *
Love Withdrawal	1.539 (0.523)	1.556 (0.542)	0.090	1.479 (0.551)	1.520 (0.595)	0.462
Disrespect	1.516 (0.413)	1.477 (0.426)	0.776	1.487 (0.458)	1.440 (0.446)	0.940
Shame	1.402 (0.608)	1.353 (0.623)	0.571	1.376 (0.629)	1.338 (0.578)	0.336

Note: * *p* < 0.05 (2-tailed).

**Table 3:** Levels of benefit perception and risk-taking across school levels.

Variables	Mean (SD)		F
	High School	College	
Benefit Perception	1.642 (1.104)	1.699 (0.937)	0.263
Risk-Taking Behavior			
Risky Driving	1.489 (0.998)	1.985 (1.274)	17.943 **
Alcohol Use	1.772 (1.114)	2.317 (1.297)	19.141 **
Drug Use	1.077 (0.631)	1.064 (0.435)	0.048
Smoking	1.299 (1.176)	1.340 (1.327)	0.100
Unprotected Sex	1.118 (0.834)	1.667 (1.551)	19.666 **

Note: ** *p* < 0.01 (2-tailed).

**Table 4:** Bivariate correlations for maternal control and study variables (above diagonal: high school; below diagonal: college).

	1.	2.	3.	4.	5.	6.	7.	8.	9.	10.
1. GEN		−0.010	0.023	0.018	−0.060	0.004	0.011	−0.009	−0.107	−0.072
2. MCVE	−0.013		0.603 **	0.461 **	0.354 **	0.497 **	0.473 **	0.292 **	0.039	0.117
3. MIF	0.114	0.482 **		0.521 **	0.415 **	0.491 **	0.553 **	0.380 **	0.142 *	0.122
4. MPA	0.051	0.424 **	0.405 **		0.393 **	0.457 **	0.648 **	0.455 **	0.116	0.087
5. MGI	−0.081	0.245 **	0.273 **	0.314 **		0.447 **	0.402 **	0.398 **	0.105	0.114
6. MLW	0.024	0.394 **	0.406 **	0.401 **	0.404 **		0.530 **	0.369 **	0.141 *	0.113
7. MD	0.133	0.567 **	0.506 **	0.522 **	0.325 **	0.595 **		0.552 **	0.220 **	0.197 **
8. MS	0.058	0.425 **	0.380 **	0.320 **	0.385 **	0.425 **	0.545 **		0.212 **	0.167 *
9. BP	−0.211 **	0.295 **	0.066	0.164 *	0.107	0.187 *	0.212 **	0.219 **		0.597 **
10. RB	−0.148	0.248 **	0.176 *	0.151	0.201 *	0.321 **	0.287 **	0.270 **	0.460 **	

Note: * *p* < 0.05, ** *p* < 0.01 (2-tailed). GEN: gender (1 = male, 2 = female); M: maternal; CVE: constraining verbal expression; IF: invalidating feeling; PA: personal attack; GI: guilt induction; LW: love withdrawal; D: disrespect; S: shame; BP: benefit perception; RB: risk-taking behavior (average score of risky driving, alcohol use, drug use, smoking, and unprotected sex).

**Table 5:** Bivariate correlations for paternal control and study variables (above diagonal: high school; below diagonal: college).

	1.	2.	3.	4.	5.	6.	7.	8.	9.	10.
1. GEN		−0.095	0.027	−0.092	−0.040	−0.086	−0.085	−0.091	−0.107	−0.072
2. PCVE	−0.127		0.534 **	0.530 **	0.390 **	0.411 **	0.503 **	0.292 **	0.102	0.155 *
3. PIF	−0.110	0.627 **		0.439 **	0.441 **	0.476 **	0.497 **	0.200 **	0.049	0.137 *
4. PPA	−0.164 *	0.544 **	0.498 **		0.414 **	0.566 **	0.620 **	0.441 **	0.095	0.128
5. PGI	−0.235 **	0.290 **	0.299 **	0.290 **		0.529 **	0.494 **	0.399 **	0.092	0.106
6. PLW	−0.093	0.462 **	0.541 **	0.582 **	0.332 **		0.643 **	0.414 **	0.162 *	0.154 *
7. PD	−0.125	0.551 **	0.551 **	0.615 **	0.382 **	0.671 **		0.521 **	0.105	0.225 **
8. PS	−0.086	0.293 **	0.343 **	0.385 **	0.372 **	0.458 **	0.446 **		0.118	0.232 **
9. BP	−0.211 **	0.074	0.156	0.321 **	0.126	0.324 **	0.277 **	0.165 *		0.597 **
10. RB	−0.148	0.130	0.039	0.166 *	0.205 *	0.262 **	0.170 *	0.113	0.460 **	

Note: * *p* < 0.05, ** *p* < 0.01 (2-tailed). GEN: gender (1 = male, 2 = female); P: paternal; CVE: constraining verbal expression; IF: invalidating feeling; PA: personal attack; GI: guilt induction; LW: love withdrawal; D: disrespect; S: shame; BP: benefit perception; RB: risk-taking behavior (average score of risky driving, alcohol use, drug use, smoking, and unprotected sex).

**Table 6:** Summary of the mediation models. Note: The coefficients are unstandardized parameter estimates.

Maternal Model	Benefit Perception				Risk-Taking Behavior			
	b	SE	*p*	CI	b	SE	*p*	CI
Psychological Control	0.054	0.017	0.001	[0.021, 0.088]	0.025	0.011	0.022	[0.004, 0.047]
Benefit Perception					0.294	0.034	<0.001	[0.227, 0.361]
Constant	1.807	0.322	<0.001	[1.174, 2.439]	1.465	0.216	<0.001	[1.040, 1.889]
	F(3, 356) = 6.104, *p* < 0.001, R^2^ = 0.049				F(4, 355) = 31.452, *p* < 0.001, R^2^ = 0.262			
Paternal Model	Benefit Perception				Risk-Taking Behavior			
	b	SE	*p*	CI	b	SE	*p*	CI
Psychological Control	0.039	0.018	0.029	[0.004, 0.073]	0.022	0.011	0.047	[0.0003, 0.043]
Benefit Perception					0.302	0.033	<0.001	[0.237, 0.367]
Constant	1.947	0.351	<0.001	[1.256, 2.638]	1.364	0.225	<0.001	[0.921, 1.807]
	F(3, 345) = 4.370, *p* = 0.005, R^2^ = 0.037				F(4, 344) = 31.705, *p* < 0.001, R^2^ = 0.269			

## Data Availability

The data that support the findings of this study are available from the corresponding author upon reasonable request.
